# Representative volume element to estimate buckling behavior of graphene/polymer nanocomposite

**DOI:** 10.1186/1556-276X-7-515

**Published:** 2012-09-20

**Authors:** Avinash Parashar, Pierre Mertiny

**Affiliations:** 1 University of Alberta, 4-9 Mechanical Engineering Building, Edmonton, Alberta, T6G 2G8, Canada

**Keywords:** Multiscale model, Nanocomposite, Buckling, Finite element method

## Abstract

The aim of the research article is to develop a representative volume element using finite elements to study the buckling stability of graphene/polymer nanocomposites. Research work exploring the full potential of graphene as filler for nanocomposites is limited in part due to the complex processes associated with the mixing of graphene in polymer. To overcome some of these issues, a multiscale modeling technique has been proposed in this numerical work. Graphene was herein modeled in the atomistic scale, whereas the polymer deformation was analyzed as a continuum. Separate representative volume element models were developed for investigating buckling in neat polymer and graphene/polymer nanocomposites. Significant improvements in buckling strength were observed under applied compressive loading when compared with the buckling stability of neat polymer.

## Background

In the recent past, graphene has emerged as a potential candidate for developing nanocomposites with improved properties [1,2]. The experimental characterization of graphene/polymer nanocomposites is a challenging process, and hence, computational approaches for predicting the behavior of such materials have also extensively been employed. Various multiscale models are available in the literature for predicting the properties of carbon nanotube (CNT)-based nanocomposites [3-5], but very few models have been presented to study graphene nanocomposites. For example, Cho et al. [6] developed a numerical model in conjunction with Mori-Tanaka approach to study the elastic constants of randomly distributed graphene in polymer. Awasthi and his team [7] investigated the load transfer mechanism between polyethylene and graphene sheets. Montazeri and Tabar [8] developed a finite element (FE)-based multiscale model to investigate the elastic constants of graphene-based nanocomposites.

Buckling in isolated graphene sheets was modeled by several researchers [9-11]. However, buckling stability of graphene/polymer nanocomposites was only reported by Rafiee et al. [12]. Using an experimental and analytical approach, up to 50% and 32% improvement in the buckling stability of nanocomposites was reported respectively. In the analytical approach, an Euler buckling formulation was employed, and elastic properties required in the Euler equation were estimated by experimental means. The discrepancies between the two buckling stabilities were attributed to scaling issues.

It is well established that the reinforcement of polymer with graphene increases the elastic modulus of the material which further improves buckling stability. The aim of this study is to propose a numerical model which can estimate the increase in buckling stability with different volume fractions of graphene and can further be extended to complex shapes and structures.

It has been reported that achieving a uniform dispersion of two-dimensional graphene sheets in polymer is more challenging compared to the mixing of one-dimensional CNT. Moreover, the application of nanocomposites is not limited to simple structures, and the comprehension of material behavior in complex structures is restricted when employing experimental and analytical methods. Consequently, research efforts are increasingly focused on numerical approaches. To overcome some of the limitations that exist in experimental and analytical work, a multiscale representative volume element (RVE) is proposed in this paper to investigate buckling phenomena in graphene/polymer nanocomposites under the assumption that graphene is uniformly distributed in the polymer. To the knowledge of the present authors, no numerical model has been reported yet to study the effect of graphene on the buckling strength of nanocomposites. In the proposed technique, graphene was modeled in the atomistic scale, whereas polymer deformation was analyzed as a continuum.

## Methods

### Finite element modeling of RVE

In this paper a finite element technique was employed in conjunction with molecular and continuum mechanics to simulate buckling in graphene/polymer nanocomposites. In the proposed RVE, the polymer, epoxy in this case, was modeled as a continuum phase whereas the deformation in graphene was evaluated using an atomistic description. Nonbonded interactions were considered as the load transfer mechanism or interphase between polymer and graphene. FE modeling was performed in the ANSYS (Version 13) software environment (ANSYS Inc., Canonsburg, PA, USA).

#### Atomistic model for graphene

To model graphene in the proposed RVE, it was assumed that graphene behaves like a space frame structure in which the covalent bonding between C-C atoms constitutes the load-bearing element while atoms act like a joint. A molecular-mechanics-based approach was employed to estimate properties for graphene in the atomistic scale. In the molecular form, potential energy of graphene can be represented as

(1) $$\sum V_{\mathrm{graphene}}=\sum V_{\mathrm{nonbonded}}+\sum V_{\mathrm{bonded}}$$

In Equation 1*V*_bonded_ represents the bonded interaction between atoms and is considered primarily responsible for maintaining the structural integrity. The potential energy term ‘*V*_nonbonded_’ includes electrostatic forces, e.g., van der Waals forces, which are considered weak in nature and can generally be neglected in comparison to bonded interactions. Li and Chou [13] established a correlation between interatomic molecular potential energies and corresponding strain energies of a beam element as described by Equations 2, 3, and 4.

(2) $$\frac{EA}{L}=K_{R}$$

(3) $$\frac{EI}{L}=K_{\theta }$$

(4) $$\frac{GJ}{L}=K_{\tau }$$

where *K*_*r*_ (938 kcalmol^−1^Å^−2^), *K*_*θ*_ (126 kcalmol^−1^rad^−2^), and *K*_*τ*_ (40 kcalmol^−1^rad^−2^) are the bond stretching, bond bending, and bond torsional resistance force constants. *E* represents the Young's modulus; *A*, the cross sectional area; *I*, the moment of inertia; *G*, the shear modulus; *J*, the polar moment of inertia of the beam element, respectively. BEAM4 elements were used in the ANSYS software environment to model graphene by connecting nodes, and material properties for those elements were estimated with the help of Equations 2, 3, and 4.

#### Continuum model for polymer

The volume fraction of graphene in polymer ranges commonly up to 10%. Most of the material volume is therefore occupied by polymer. Simulating the polymer phase on the atomistic scale would require large efforts in dealing with large numbers of degrees of freedom as well as substantial computational cost. Therefore, as a reasonable compromise, the polymer phase was modeled as a continuum, and three-dimensional SOLID45 elements were used for meshing the geometry. Epoxy with a Young's modulus of 3.4 GPa and a Poisson’s ratio of 0.42 was considered as the polymer material in the present work.

#### Interphase between graphene and polymer

**Figure 1:**
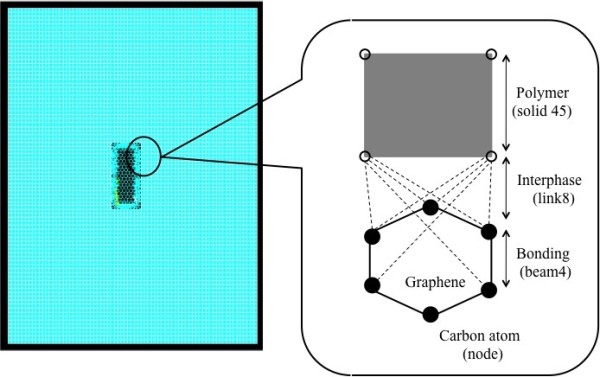
Schematic of multiscale model.

In this research paper, nonbonded interactions were considered as load transfer mechanisms between the polymer and graphene. Naturally, load transfer between nonfunctionalized graphene sheets and polymer takes place through van der Waals interactions. The Lennard Jones ‘6-12’ potential given in Equation 5 was employed to estimate the properties for the interface region, which is depicted in the schematic in Figure 1.

(5) $$U\left(r\right)=4\gamma \left[{\left(\frac{\phi }{r}\right)}^{12}+{\left(\frac{\phi }{r}\right)}^{6}\right]$$

where *r* is the atomistic distance between atoms, *φ* (0.34 nm) is the hard sphere radius, and *γ* (0.556 kcal/mole) is the potential well depth. In the finite element environment, these nonbonding interactions were modeled with the help of LINK8 truss elements. As shown in Equation 6, properties for those truss elements were estimated by comparing the classical continuum strain energy of truss elements with the Lennard Jones ‘6-12’ potential. Note that the truss model described herein was earlier employed by Li and Chou [14] to simulate the interaction between CNT and epoxy polymer.

(6) $$E\left(r\right)=\frac{8\gamma R_{\mathrm{Eq}}}{A{\left(r-R_{\mathrm{Eq}}\right)}^{2}}\left[{\left(\frac{\phi }{r}\right)}^{12}+{\left(\frac{\phi }{r}\right)}^{6}\right]$$

where *R*_Eq_ is the initial undeformed length, *r* is the deformed length, *A* is the cross sectional area, and *E*(*r*) is the Young's modulus of the truss element.

#### Eigenvalue buckling analysis

A linear analysis for mode one buckling was performed in this work, which is associated with the computation of a bifurcation load and corresponding buckling mode. The analysis in the finite element environment was divided into two sections, i.e., a pre-buckling and a post-buckling analysis [15].

#### Pre-buckling analysis

The pre-buckling analysis was performed in the finite element environment to compute the reference stresses S* within each element. These computed reference values are then used for estimating the geometric stiffness matrix *K*_*G*_, which is needed in the post-buckling analysis. To initiate the analysis in the finite element model, the general matrix form equation provided in Equation 7 was employed to estimate nodal displacements [*Q*] when the structure is subjected to the reference unit load *P*_*R*_. In Equation 7 the total stiffness matrix [*K*_*0*_] is the assembled global stiffness matrix for the multiscale structure composed of beam, truss, and solid elements. Conventional assembly techniques can be used to obtain the global stiffness matrix.

(7) $$\left[K_{0}\right]\left[Q\right]=\left[P_{R}\right]$$

Equation 8 represents the general solution that is obtained in the finite element analysis after solving the assembled matrices defined in Equation 7.

(8) $$\left\{Q*\right\}={\left[K_{0}\right]}^{-1}\left[P_{R}\right]$$

The nodal displacement vectors {Q*} obtained from Equation 8 are further post-processed to compute strains and corresponding stress values S* within each element using Equation 9, in which matrix [*D*] represents the elasticity matrix (material property) while matrix [*B*] is a function of approximating polynomial or shape functions.

(9) $$\left\{S*\right\}=\left[D\right]\left[B\right]\left\{Q*\right\}$$

#### Post-buckling analysis

The post-buckling analysis corresponds to a general eigenvalue problem as defined by Equation 10, in which *λ* and *v* are the load factor and the eigenvector of displacements. The geometric stiffness matrix *K*_*G*_ used in Equation 10 is defined with the help of Equation 11, where Γ is composed of derivatives of shape functions and *S* is a function of the stress S* estimated in the pre-buckling analysis.

(10) $$\left[K_{0}+\lambda K_{G}\right]\nu =0$$

(11) $$\left[K_{G}\right]=\int_{V}\Gamma^{T}S\Gamma dV$$

By solving Equation 10, the lowest possible value for the load factor *λ*_min_ can be determined, which is then employed to estimate the buckling force given by Equation 12.

(12) $$Buckling\;force=\lambda_{\min}\times Reference\;load$$

## Results and discussion

The proposed RVE was employed to understand the buckling behavior of graphene in a polymer matrix when graphene was assumed to be uniformly distributed. A second separate RVE structure with equal dimensions was developed using finite element modeling to study the buckling in neat (homogenous) polymer.

**Figure 2:**
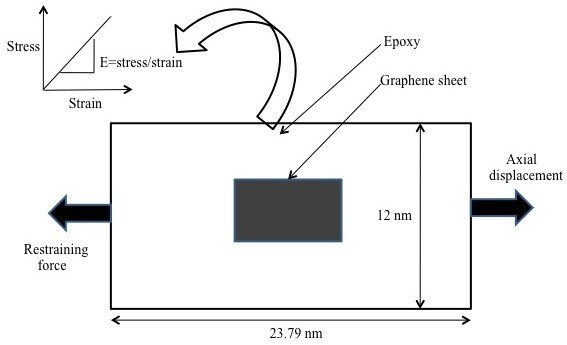
Schematic of model and boundary conditions for estimating Young's moduli of developed RVE.

**Figure 3:**
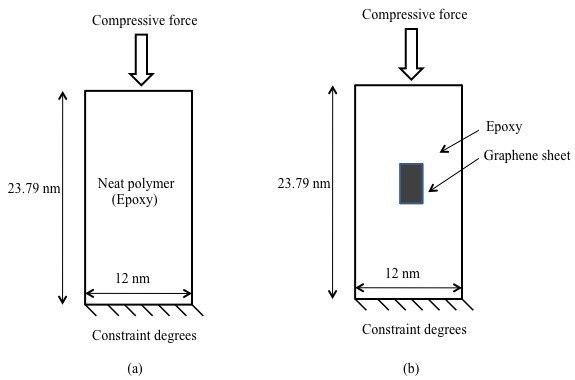
Boundary conditions and dimensions for (a) neat polymer model and (b) multiscale graphene/polymer model.

To validate the results of the proposed RVE, an alternative method (indirect method) was also employed in this paper. The proposed RVE model with boundary conditions as shown in Figure 2 was first used to estimate the Young's modulus of the RVE as a whole for various volume fractions of graphene. The estimated material properties (*E*) were then used in defining the homogenous rectangular plate with dimensions of the proposed RVE model. The buckling loads were calculated for this homogenous rectangular plate shown in Figure 3a and were compared with the direct approach defined in Figure 3b.

The analytical formulation to estimate the buckling load for a rectangular plate is given in Equation 13, where *P*_*x*_ is the applied unidirectional compressive load, *w* is the displacement in the outward normal direction, and *D* is flexural rigidity (i.e., a function of the Young's modulus). Boundary conditions were kept identical in all RVE models. Only the value of flexure rigidity was varied, which afforded the proposed RVE model the capability of yielding *E* for different graphene volume fractions.

(13) $$D\nabla^{4}w\left(x,y\right)+P_{x}\frac{\partial^{2}w}{\partial^{2}x}=0$$

The boundary conditions along with dimensions for the proposed RVE model are shown in Figure 3. The thickness of graphene in the atomistic scale and for epoxy as a continuum phase was kept constant at 0.344 nm, whereas the thickness of the interphase was kept at 0.172 nm according to [14]. The graphene volume fraction in the proposed RVE model was varied by changing the size of the graphene sheet, leading to graphene volume fractions ranging from 2% to 6%. Filler volume fractions of reasonable and practical magnitude were thus studied, omitting the agglomeration effects in graphene nanocomposites with high filler content.

**Figure 4:**
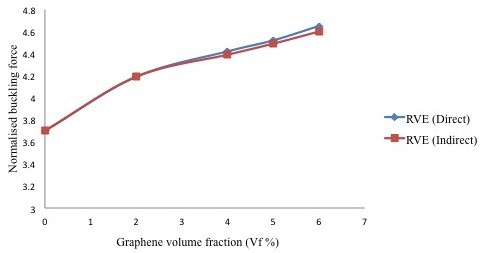
**Normalized buckling force estimated from multiscale modeling for graphene/polymer nanocomposites.** Ordinate data was normalized by dividing the critical buckling force by 10-2 nN.

The results obtained from the developed RVE structures were plotted in Figure 4. These data show a significant improvement in the buckling performance of nanocomposites under compressive loading. The buckling strength of neat epoxy was herein considered as the reference level. Buckling strengths that are calculated from the direct and indirect approach are in good agreement, which validates the proposed numerical technique. In the current work, up to 26% improvement in the buckling strength of epoxy was estimated for only 6% volume fraction of graphene.

## Conclusions

In this study a representative volume element method was successfully employed to investigate the buckling phenomenon in graphene/polymer nanocomposites, where graphene was assumed to be uniformly distributed. Graphene was modeled in the atomistic scale and polymer as a continuum. A significantly enhanced buckling strength of graphene reinforced polymers was observed as compared to neat polymer, i.e., buckling strength of graphene/polymer nanocomposite improved by 26% with only 6% filler volume fraction.

## Competing interests

The authors declare that they have no competing interests.

## Authors' contributions

Both authors made equally valuable contributions to this paper. Both authors read and approved the final manuscript.
